# Editor's Letter Box

**Published:** 1892-07-30

**Authors:** Charles W. Macara

**Affiliations:** St. Anne's-on-the-Sea, Chairman of the Manchester and Salford "Life-boat Saturday" Committee


### Editor's Letter Box.

[Our correspondents are reminded that prolixity of statement is a great bar to publication, and that brevity of style and conciseness of statement greatly facilitate early insertion.]

Sir,—It is estimated that 17,000 of the bravest of Britain's sons are ever ready at the call of duty to man the fleet of upwards of 300 boats which have been stationed by the Royal National Life-boat Institution around our treacherous coasts.

Nearly 37,000 lives have been saved by this great organisation, which has been supported entirely by voluntary contributions since it was established in 1824. No fewer than 379 men, women, and children were rescued from one ship alone this year, viz., the German liner "Eider," which went on the rocks off the Isle of Wight during a fog. In recognition of this service, His Imperial Majesty the Emperor of Germany, in addition to gifts to the various coxswains who effected this rescue, gave a donation of £200 to the Institution in the welfare of which he takes the greatest interest.

A considerably larger income than that of last year is required to maintain this service efficiently, and also to provide for the rapid increase in the number of boats which has taken place of late years. It is now well known that in 1890 the income from subscriptions, donations, and dividends was utterly inadequate for the greatly increasing requirements, and necessitated a large draft from the reserve fund, which was, and still is, much too small for an institution with operations of such magnitude, especially taking into consideration the numerous contingencies that have continually to be met and the renewal of obsolete boats. This renewal has been suspended of late as far as possible, till it had been decided by the recent experiments at Lowestoft which was the best type of lifeboat for the various parts of the coast.

Admiral H.R.H the Duke of Edinburgh, speaking at the opening of the new dock at Preston in June last, said:—

I would allude to another point also, which is connected with the sea. It is that I received an invitation to go to St. Anne's to-day to take part in a lifeboat demonstration. I regret, however, that I was not able to accept the invitation, as the programme of to-day's proceedings would not admit of it. But I think I would like to say one word, being myself associated with the National Lifeboat Institution, as to the great and valuable work which is done by that Institution. In the year 1890 the contributions from Lancashire and the surrounding counties did not reach the sum of £3,000, but owing to special exertions which have been made during the last year the contributions from the counties I have named have reached nearly £21,000. In that respect Lancashire and the neighbouring counties have shown an example which I hope will be followed by all the other counties in this kingdom.

"Life-boat Saturday" in Manchester and Salford in October last resulted in a sum of £5,500 being raised, and it was unanimously decided that this collection is to be an annual event.

Liverpool, Bolton, Oldham, and Stockport have made similar efforts successfully.

The press gave invaluable help by advocating the cause, and several editors opened their columns to receive subscriptions, which resulted in a large sum being raised.

Sir Edward Birkbeck, chairman of the Institution, has rendered immense service towards the saving of life at sea by his recent successful motion in the House of Commons with reference to the provision of telegraphic or telephonic communication between all signal, coastguard, and lifeboat stations round the coast.

All that is now wanted is that "Life-boat Saturday" should become general throughout the country, as by this means an opportunity is afforded everyone to contribute—no matter how small the sum may be—to the support of an Institution which is one of the grandest monuments of England's greatness.

The Manchester and Salford "Life-boat Saturday" Committee earnestly hope they will be joined this year by many other towns where this popular movement has not yet been taken up, so that the income of the Royal National Life-boat Institution, which last year showed an increase of about £23,000 over 1890, will be still further largely increased, which is absolutely necessary if this great life-saving service is to be maintained efficiently and extended in accordance with the ever growing requirements of the greatest maritime nation of the world.

Charles W. Macara,

St. Anne's-on-the-Sea, Chairman of the Manchester and Salford "Life-boat Saturday" Committee.

